# Supplementary material for: Structural basis of malodour precursor transport in the human axilla
Source: eLife. 2018 Jul 3;7:e34995. doi: 10.7554/eLife.34995 (PMC6059767; doi:10.7554/eLife.34995)
Supplement: Supplementary file 1. [file elife-34995-supp1.docx]

**Supplementary file 1.**

**Table S1 List of strains used in this study**

| Strain | Genotype | Reference |
| --- | --- | --- |
| BW25113 | F-, Δ(*araD*-*araB*)567, Δl*acZ*4787(::*rrnB*-3), lambda-, rph-1, Δ(*rhaD*-*rhaB*)568, *hsdR*514 | Baba *et al.* (2006) |
| JW1237 | *ΔoppC::kan^R^* | Baba *et al.* (2006) |
| JW3511 | *ΔdppC::kan^R^* | Baba *et al.* (2006) |
| JW1626 | *ΔdtpA::kan^R^* | Baba *et al.* (2006) |
| JW3463 | *ΔdtpB::kan^R^* | Baba *et al.* (2006) |
| JW4091 | *ΔdtpC::kan^R^* | Baba *et al.* (2006) |
| JW0699 | *ΔdtpD::kan^R^* | Baba *et al.* (2006) |
| JW3686 | *ΔtnaA::kan^R^* | Baba *et al.* (2006) |
| JW2975 | *ΔmetC::kan^R^* | Baba *et al.* (2006) |
| JW1614 | *ΔmalY::kan^R^* | Baba *et al.* (2006) |
| JW1285 | *ΔsapC::kan^R^* | Baba *et al.* (2006) |
| JW0590 | *ΔcstA::kan^R^* | Baba *et al.* (2006) |
| *ΔoppC* | *ΔoppC* | This study |
| *ΔdppC* | *ΔdppC* | This study |
| *ΔdtpA* | *ΔdtpA* | This study |
| *ΔdtpB* | *ΔdtpB* | This study |
| *ΔdtpC* | *ΔdtpC* | This study |
| *ΔdtpD* | *ΔdtpD* | This study |
| *ΔtnaA* | *ΔtnaA* | This study |
| ΔDB1 | *ΔoppC ΔdppC* | This study |
| ΔDB5 | *ΔdtpA ΔdtpB ΔdtpC ΔdtpD* | This study |
