# Supplementary material for: Structural basis of malodour precursor transport in the human axilla
Source: eLife. 2018 Jul 3;7:e34995. doi: 10.7554/eLife.34995 (PMC6059767; doi:10.7554/eLife.34995)
Supplement: Supplementary file 2. [file elife-34995-supp2.docx]

**Supplementary file 2.**

**List of plasmids used in this study**

| Plasmid | Size | Description | | Reference |
| --- | --- | --- | --- | --- |
| pCP20 | 9.40 | | FLP^+^, pSC101 origin, λ cI857+, λ p_R_ Rep^ts^, Amp^R^, Cm^R^ | Cherepanov & Wackernagel (1995) |
| pKD46 | 6.33 | | Contains Kan^R^ cassette | Datsenko & Wanner (2000) |
| pBADcLIC2005 | 4.14 | | pBAD vector modified with LIC cassette, P_BAD_ | Geertsma & Poolman (2007) |
| pDtpA | 5.64 | | *dtpA* in pBADcLIC | This study |
| pDtpB | 5.61 | | *dtpB* in pBADcLIC | This study |
| pDtpC | 5.60 | | *dtpC* in pBADcLIC | This study |
| pDtpD | 5.62 | | *dtpD* in pBADcLIC | This study |
| pSH0415 | 5.33 | | *SH0415*  in pBADcLIC | This study |
| pSH1446 | 5.65 | | *SH1446*  in pBADcLIC | This study |
