# Supplementary material for: Structural basis of malodour precursor transport in the human axilla
Source: eLife. 2018 Jul 3;7:e34995. doi: 10.7554/eLife.34995 (PMC6059767; doi:10.7554/eLife.34995)
Supplement: Supplementary file 3. [file elife-34995-supp3.docx]

**Supplementary file 3.**

**List of oligonucleotides used in this study**

| Primer Name | Sequence 5-3 |
| --- | --- |
| Keio Mutants | |
| *dppC* F | TGCTAATCGACACCGC |
| *dppC* R | TGGCGATCATCACGCG |
| *oppC* F | GCTGGCGTTATTGCCG |
| *oppC* R | CGCATCGAGCATCCGC |
| *dtpA* F | GACCTACAAAACATTACACTGGC |
| *dtpA* R | TAACGGCAAAGTAATCGTCACCG |
| *dtpB* F | AGCGTAAACACCTTATCTGGC |
| *dtpB* R | TGATATCTATGCAGGTACGCC |
| *dtpC* F | GTGTGAAATCGGCGCTCACTATCCG |
| *dtpC* R | TAGCGTAGATAAAGAGACAGATCGG |
| *dtpD* F | CTCGCCAGTTCGTCGATAGCC |
| *dtpD* R | GCGTGAACGCCTTATCCAGCC |
| *tnaA* F | TTACTTGTTTTAGTAAATGATGG |
| *tnaA* R | TCAGCTTGATCAGTGATGATGCC |
| pBAD Overexpression | |
| *dtpA* pBADcLIC-F | ATGGGTGGTGGATTTGCTGTGTCCACTGCAAACC |
| *dtpA* pBADcLIC-R | TTGGAAGTATAAATTTTCCGCTACGGCTGCTTTC |
| *dtpB* pBADcLIC-F | ATGGGTGGTGGATTTGCTATGAATACAACAACACCCATGGGG |
| *dtpB* pBADcLIC-R | TTGGAAGTATAAATTTTCATGGCTTTCCGGCGTCG |
| *dtpC* pBADcLIC-F | ATGGGTGGTGGATTTGCTATGAAAACACCCTCACAGCC |
| *dtpC* pBADcLIC-R | TTGGAAGTATAAATTTTCATCGTTGCTCTCCTGTATC |
| *dtpD* pBADcLIC-F | ATGGGTGGTGGATTTGCTATGAATAAACACGCATCACAGCCG |
| *dtpD* pBADcLIC-R | TTGGAAGTATAAATTTTCAGACTCCAGCGCCAGC |
| *0415* pBADcLIC-F | ATGGGTGGTGGATTTGCTATGAAGAAGCACAGTAAAGATTAC |
| *0415* pBADcLIC-R | TTGGAAGTATAAATTTTCATTTTTAATTGCTGTTTTAAAATATAAACC |
| *1446* pBADcLIC-F | ATGGGTGGTGGATTTGCTATGGCAACAAATAACTCCCAT |
| *1446* pBADcLIC-R | TTGGAAGTATAAATTTTCGTGAATACCTTTCATAGCTTTCGT |
| Site Directed Mutagenesis of SH1446* | |
| Y41A-F | ATTCAGTTACgccGGTATGCGTG |
| Y41A-R | CTTTCCCAAAACTCTACG |
| Y41F-F | ATTCAGTTACttcGGTATGCGTG |
| Y41F-R | CTTTCCCAAAACTCTACG |
| N167A-F | TATGTCTGTTgccTTAGGTGCATTAATTTC |
| N167A-R | TAGAAAATAACGAAACCTGC |
| N347A-F | CCAATCTATCgctCCATTATTTATTTTATTATTTGC |
| N347A-R | ACCCAGTTAACTGAGAATTG |
| Y411W-F | TATTTTATCAtggGTAATCTGTGTTATTGG |
| Y411W-R | ACCCAGTTAACTGAGAATTG |
| Y411A-F | TATTTTATCAgctGTAATCTGTGTTATTGGG |
| Y411A-R | ACCCAGTTAACTGAGAATTG |
| *Mutations are underlined in primer sequences | |
